# Poor sleep quality and suicide attempt among adults with internet addiction: A nationwide community sample of Korea

**DOI:** 10.1371/journal.pone.0174619

**Published:** 2017-04-06

**Authors:** Kiwon Kim, Haewoo Lee, Jin Pyo Hong, Maeng Je Cho, Maurizio Fava, David Mischoulon, Dong Jun Kim, Hong Jin Jeon

**Affiliations:** 1 Department of Psychiatry, Depression Center, Samsung Medical Center, Sungkyunkwan University School of Medicine, Seoul, Korea; 2 Department of Psychiatry, Seoul Medical Center, Seoul, Republic of Korea; 3 Department of Psychiatry and Behavioral Sciences, Seoul National University College of Medicine, Seoul, Korea; 4 Depression Clinical and Research Program, Massachusetts General Hospital, Harvard Medical School, Boston, United States of America; 5 Department of Health Sciences & Technology, Samsung Biomedical Research Institute (SAIHST), Sungkyunkwan University, Seoul, Korea; Chiba Daigaku, JAPAN

## Abstract

**Purpose:**

Internet addiction (IA) is defined as a psychological dependence on the internet, regardless of the type of activities once logged on, and previous studies have focused on adolescents and young adults. The aim of this study was to investigate the association between suicide attempts and sleep among community-dwelling adults with IA.

**Methods:**

The Young’s Internet Addiction Test (IAT), the Korean version of the Composite International Diagnostic Interview (K-CIDI) and a suicide questionnaire were used in this cross-sectional multistage, cluster sampling population-based study. A total of 3212 adults aged 18–64 years were interviewed face-to-face, and they had been randomly selected through a one-person-per-household method.

**Results:**

Of the 3212 adults, 204 were assessed as having IA (6.35%). Adults with IA were younger, and more frequently male, unmarried, and unemployed, and had poorer sleep quality than adults without IA (32.8% vs. 19.8%), whereas there was no significant difference in the absolute duration of sleep between the two groups. Adults with IA showed more frequent difficulty initiating and maintaining sleep, non-restorative sleep, daytime functional impairment, and duration of sleep more than 10 hours on weekdays than adults without IA. IA with poor sleep quality was significantly associated with lifetime suicide attempts (AOR = 3.34, 95% CI 1.38–8.05) after adjusting for demographic covariates. Adults with IA who had more sleep problems showed more severe IA, especially those who experienced a previous suicidal attempt. Among mental disorders, IA with poor sleep quality was significantly associated with anxiety disorder and overall psychiatric disorders.

**Conclusions:**

Among adults with IA, poor sleep quality was found to be associated with more severe IA and lifetime suicide attempt.

## Introduction

The Internet has become an essential part of our lives, with multiple applications being available on the desktop computer, laptops, tablet devices, and a smart watch linked to each smartphone. This phenomenon has led to the ambiguous boundary of ‘off-line’ for users [1]. Internet addiction (IA) is defined as a psychological dependence on the internet, irrespective of the type of activity once logged on. The overall prevalence of IA varies from 0.3% [2] to 8% in adolescents and it is reported to be up to 20% among adults [3].

Epidemiologic data on IA is dependent on its definition as ‘Internet addiction’ or ‘Problematic internet use’ and a higher prevalence has been depicted in countries where accessibility and technology are widespread [2]. Leaving aside its direct association with disruption in work and social relationships along with a negative affect resulting from being off-line [4], IA shows multiple associations with psychiatric comorbidities and general health- related behaviors [3, 5, 6]. Considering its influence on the society, DSM-5 proposed the inclusion of ‘internet gaming disorder’ in section III, but more research related to this domain is sorely needed [7].

Many studies have reported sleep disturbances related to IA in the adolescent and young adult group, more disrupted sleep-wake schedule [8] and a higher rate of insomnia [9, 10] along with other specific psychiatric disorders, such as drug and alcohol use disorder [11], gambling disorder [12], depression [13, 14], anxiety disorders [13–15], and attention-deficit hyperactivity disorder [16]. An association between sleep problems and suicidal behaviors has been widely documented [17–19]. But systematic analyses of insomnia and suicide-related thoughts and behaviors are still inconsistent across studies and there is a lack of empirical studies focusing on mediating factors in the insomnia and suicide relationship [20]. Similar to these numerous studies on sleep problems and suicidality in the last 10 years, the negative impact of internet use as an internet addiction has been continuously researched [2, 21–24].

Although there are findings showing functional changes in cognitive control in samples with IA representing impulsivity [25, 26] and protective function of restorative sleep in suicidal behaviors [27], the question concerning features related to sleep problems and suicidal behaviors, especially in internet addiction, remains unanswered. The aim of the current study was to investigate the association between suicide attempts and sleep problems among community-dwelling adults with IA. We hypothesized that IA would be associated with sleep problems, and that adults with both IA and sleep problems would show more impulsivity in suicide-related behaviors. In a sample from a cross-sectional national study, we compared the sleep quality profiles and suicide-related profiles between internet addicts and non-addicts, and evaluated their association.

## Methods

### Study population

Participants were recruited from the Korean Epidemiologic Catchment Area study Replication (KECA-R), a nationwide survey conducted between August 1, 2006 and April 30, 2007, and the detailed design has been documented elsewhere [28]. All eligible residents aged 18–64 years, listed in the updated 2005 population census at community register offices were included in the target population. Multistage, cluster sampling design was adopted. The Institutional Review Board of the Seoul National University College of Medicine approved this study. All subjects were fully informed about the aims and methods of the survey prior to interview completion and written informed consent was obtained prior to participation, as these procedures were approved by IRB. Interviews were conducted on a face-to-face basis. From among this population, 3,212 participants completed the interview regarding IA (Response rate 81.7%). A total of 3,198 participants completed the interview related to the sleep measure.

### Interviewer training

A total of 78 interviewers, who were experts in conducting psychiatric epidemiologic surveys, were recruited from each catchment area. All interviewers underwent a 5-day training session that included didactic sessions concerning general interview skills, interview instruments, mock interviews, and role-playing exercises using the standard protocols and training materials developed by the World Health Organization [29, 30].

### Assessment of DSM-IV disorders

The Korean version of Composite International Diagnostic Interview (K-CIDI), a validated structured diagnostic interview instrument designed by Cho et al. according to the World Health Organization guidelines, was applied to make psychiatric diagnoses [31, 32]. DSM-IV diagnoses showed good concordance with the clinical diagnosis with blind clinical re-interviews, using the Structured Clinical Interview for the DSM-IV (SCID) (κ values between 0.50 and 1.00) [33]. The K-CIDI was also applied in the Korean Epidemiologic Catchment Area (KECA) study, conducted between June 2001 and November 2001 [34].

### The measure of internet use

The Korean version of Young's Internet Addiction Test (IAT) [35] was applied to evaluate internet use. This scale is a modified version of the IAT, which is a reliable and valid measure of addictive use of the internet designed by Dr. Kimberly Young [36]. It is composed of 20 questions with 5 Likert scales and it was validated (internal consistency, alpha = 0.942) in this study. The sum of the scores for these 20 components yields quantitative internet use (range, 0–80) with a cutoff of 50 points for IA.

### The measure of sleep

Five DSM-IV-defined sleep disturbances were assessed using the following questions: 1. “How often have you had trouble sleeping due to difficulty falling asleep since last month?”, for difficulty initiating sleep (DIS); 2. “How often have you had a difficult time sleeping because you often woke up after falling asleep since last month?”, for difficulty maintaining sleep (DMS); 3. “How often have you had trouble sleeping because you woke up too early and could not go back to sleep since last month?”, for early morning awakening (EMA); 4. “Have you ever woken up feeling tired and not refreshed since last month?”, for non-restorative sleep (NRS); and 5. “How much disturbance have you experienced due to poor sleep quality during day time activity such as fatigue, impairment in social, occupational, or other important areas of your life and mood disturbance?”, for daytime functional impairment due to poor sleep quality (DFI). For questions 1, 2, 3, and 4, answers were coded as ‘no,’ ‘once or twice a week,’ ‘three or four times a week,’ and ‘almost every night.’ For question 5, answers were coded as ‘seldom’, ‘a little disturbance’, ‘much disturbance’, and ‘severe disturbance’. Answers were recorded using a 4-point Likert scale ranging from 1 (‘no’ or ‘seldom’) and 4 (‘almost every night’ or ‘severe disturbance’). We also enquired about the duration of DIS by asking the question, “How long have you been suffering from difficulty initiating sleep (DIS)?”, and answers were coded as ‘no,’ ‘less than 6 months’, and ‘more than 6 months’. They were also recorded using a 3-point Likert scale ranging from 1 (no) to 3 (more than 6 months).

To assess sleep duration on weekdays separately from weekends and holidays, participants were asked to answer the following question: “On an average, how many hours do you sleep each night on weekdays?” and the same question was asked to evaluate sleep duration on weekends including holidays. Answers to the question related to sleep duration during the weekdays and weekends including holidays were also classified into the following three categories: ‘5 h or less,’ ‘6 to 9 h,’ and ‘10 h or more per day.’ ‘5 h or less’ was defined as a very short sleep time and ‘10 h or more per day’ was defined as a long sleep time according to the ICSD specific criteria. The presence of DIS, DMS, NRS, DFI and Duration of sleep more than 10 hours on weekdays were defined as ‘Any problem in sleep quality’. We applied description of “Without poor sleep quality” to whom answered the least items to all 6 questions as sum of total score 6. Rest of participants who reported more than total score of 6 were classified as “With poor sleep quality”. We analyzed the relationship between sleep quality and suicide attempts among participants with and without IA.

### The measure of suicidal ideation, plan and attempt

We asked all subjects about suicidal ideation, plans, and attempts by using structured questions. The questions were, “Have you ever had a serious thought about committing suicide?”, for suicidal ideation, “Have you ever made a plan for committing suicide?”, for suicidal plan, and “Have you ever attempted suicide?”, for suicide attempt [37, 38]. The participants answered these questions in a dichotomous manner as “Yes” or “No”. The age at the first suicide attempt and the number of suicide attempts were also assessed by open questions. The questions showed strong validity between psychiatrists and interviewers with inter-rater reliability expressed as kappa values from 0.74 to 1.00 and test-retest reliability of 0.84 for MDD in a preliminary study for this KECA-R [39].

### Other measures

To obtain information regarding socio-demographic variables, self-reported questionnaires from the 2006 KECA study were applied. Socio-demographic variables included gender, age, marital status, education years, occupational status, and monthly income. The participants responded to the questionnaire related to education years ranging from no education, less than 6 years, 7 to 9 years, 10 to 12 years, to more than 12 years. Answers to the question related to occupational status were divided into three categories; full time, part time, and unemployed. Monthly income was divided into three categories; less than 2000 dollars, 2000 to 3000 dollars, and more than 3000 dollars.

### Statistical analysis

Subjects were divided into two groups according to the Young’s IAT with a cutoff value of 50. The two groups were compared with respect to age, female gender proportion, education years, and duration of sleep by using Student’s t-test, and with respect to marital status, education categorization, occupational status, monthly income, and presence of suicidal ideation, plan and attempt by using the chi-square test (two-tailed) and Fisher’s exact test. Multiple logistic regression analyses were performed to analyze the correlation of IA with the dependent variable, sleep quality, after adjusting for age, sex, education years, and marital status. In the group with poor sleep quality and the group without poor sleep quality, multiple logistic regression analysis was applied after adjusting for demographic variables. To identify comorbidities of IA, multiple logistic regression analysis with adjustment for other demographic variables was used. The association between poor sleep quality and severity of IA is depicted with bar charts after dividing the subjects into 3 groups, ‘No sleep problem’ (total score 6), ‘mild to moderate sleep problem’ (total score between 7 and 13), and ‘severe sleep problem’ (total score more than 14). Statistical analysis was performed by using SPSS 21.0, and the level of significance was set at P < 0.05.

## Results

### Demographic and clinical profiles

In the 3,212 participants who completed the questionnaires regarding IA, the average score on the IAT was 28.00 ± 10.80. A total of 204 (6.35%) subjects were classified as those who had IA (whose score on the IAT was ≥ 50). Demographic and clinical profiles of the two groups are shown in Table 1. Participants with IA (IAT ≥ 50) were younger, had more education, tended to be male, unmarried and unemployed compared to those without IA (IAT score of < 50). Total participants who completed suicide interview were 3190 in ideation question(number of participants with IA:201, number of participants without IA:2989), 3188 in plan(number of participants with IA:201, number of participants without IA:2987), and 3185 in attempt(number of participants with IA:199, number of participants without IA:2986). Participants with IA showed more frequent history of suicidal ideation and plan, whereas no significant differences were found in monthly income, absolute duration of sleep, and previous suicide attempt history between the two groups.

**Table 1:** Demographic and Clinical Profiles of Adults with and without Internet Addiction in a Nationwide Community Sample of Korea (*n* = 3212).

Profiles	Internet addiction (*n* = 204)		No internet addiction (*n* = 3008)		Statistics t or χ2 p-value	
Internet Addiction Test (IAT, mean, SD)	58.95	(9.27)	25.90	(7.02)	50.0	< 0.0001
Age (mean, SD)	26.9	(8.8)	35.0	(9.9)	12.6	< 0.0001
Female gender (%)	84	41.2%	1702	56.6%	18.4	< 0.0001
Marital status (%)						
Married	44	21.6%	1876	62.6%	151.0	< 0.0001
Divorced/widowed/separated	6	2.9%	133	4.4%		
Unmarried	154	75.5%	990	33.0%		
Education years (mean, SD)	13.8	(2.6)	13.8	(2.6)	0.27	0.79
Education years (%)						
No education	2	1.0%	6	0.2%	14.99	0.005
1–6	1	0.5%	51	1.7%		
7–9	4	2.0%	90	3.0%		
10–12	54	26.5%	1067	35.5%		
12+	143	70.1%	1794	59.6%		
Occupation (%)						
Full time	55	27.0%	1452	48.3%	40.0	< 0.0001
Part time	6	2.9%	134	4.5%		
Unemployed	143	70.1%	1421	47.3%		
Monthly income (%)						
< 2000$	50	34.7%	850	33.1%	0.26	0.88
2000–3000$	45	31.3%	792	30.9%		
≥ 3000$	49	34.0%	923	36.0%		
Duration of sleep (mean, SD)						
Weekdays (hrs)	7.10	(1.65)	6.91	(1.31)	1.63	0.10
Weekends and holidays (hrs)	7.94	(1.75)	7.84	(1.64)	0.90	0.37
Suicide^a^						
Suicide ideation	45	22.4%	452	15.1%	7.56	0.006
Suicide plan	14	7.0%	79	2.6%	12.4	< 0.0001
Suicide attempt	11	5.5%	82	2.7%	5.09	0.024

SD, standard deviation. Internet addiction was defined as a score of more than 50 on the Young’s Internet Addiction Scale.

### Sleep quality and its correlation with IA

**Fig 1:**
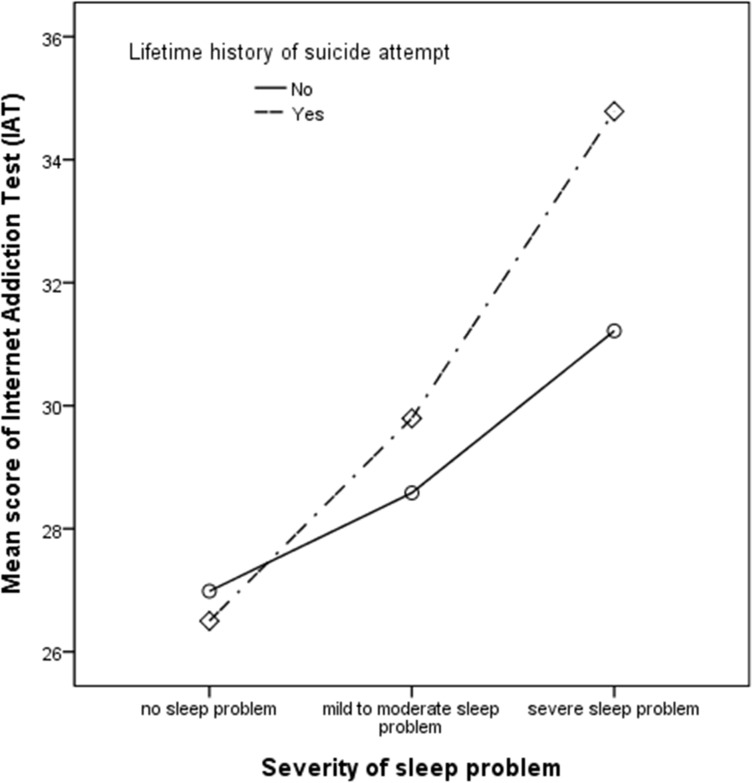
Association between poor sleep quality and severity of internet addiction in adults with and without a lifetime history of suicide attempt (*n* = 3212)

As shown in Table 2, participants with IA showed poorer sleep quality than participants without IA (AOR = 1.73, P < 0.0001) after adjusting for age, sex, education years, and marital status. Participants with IA showed a significantly higher tendency to have DIS (AOR = 1.66, P = 0.028), with a significantly longer duration shown as ‘DIS for more than 6 months’ (AOR = 1.73, P = 0.032) than participants without IA. Subjects with IA had a significantly more frequent DMS (AOR = 1.96, P = 0.006), NRS (AOR = 1.53, P = 0.017), and DFI (AOR = 1.92, P = 0.009) than subjects without IA. Although the IA group showed no significant difference in extremely long sleep time (duration of sleep more than 10 hours) on weekends and holidays, participants with IA showed a significantly longer sleep time during the weekdays (AOR = 1.99, P = 0.019) than the group without IA. No significant differences were found in early morning awakening, short sleep time (duration of sleep less than 5 hours) during the weekdays and weekends between the two groups. As shown in Fig 1, participants who had more sleep problems showed more severe IA and this tendency was found to be much stronger in the group with a previous suicidal attempt. For the questionnaires applied to measure sleep quality were based on DSM-IV Sleep Disorder criteria, they asked sleep behavior characteristics during past one month. These results should be taken careful interpretation for this temporal assessment in variables.

**Table 2:** Sleep quality among adults with and without internet addiction (*n* = 3212).

Sleep Quality profiles	Internet addiction (*n* = 204)		No internet addiction (*n* = 3008)		Statistics	
	*N*	%	*N*	%	AOR	(95% CI)
Difficulty initiating sleep (DIS)	26	12.7%	200	6.7%	1.66	(1.06–2.62)^*^
DIS for more than 6 months (DIS6)	21	10.3%	195	6.5%	1.73	(1.05–2.84)^*^
Difficulty maintaining sleep (DMS)	24	11.8%	195	6.5%	1.96	(1.22–3.14)^**^
Early morning awakening (EMA)	6	2.9%	105	3.5%	0.90	(0.38–2.11)
Non-restorative sleep (NRS)	50	24.5%	447	14.9%	1.53	(1.08–2.16)^*^
Daytime functional impairment due to poor sleep quality (DFI)	22	10.8%	145	4.8%	1.92	(1.17–3.16)^**^
Duration of sleep less than 5 hours						
Weekdays	24	12.5%	276	9.9%	1.25	(0.78–1.98)
Weekends and holidays	12	6.2%	178	6.2%	0.99	(0.53–1.87)
Duration of sleep more than 10 hours						
Weekdays	17	18.3%	182	6.0%	1.99	(1.12–3.54)^*^
Weekends and holidays	39	9.2%	160	6.0%	1.07	(0.73–1.56)
Poor sleep quality^a^	67	32.8%	597	19.8%	1.73	(1.28–2.34)^***^

^a^ Any problem such as DIS, DMS, NRS, DFI, and Duration of sleep more than 10 hours on weekdays

AOR, adjusted odds ratio; CI, confidence interval

Adjusted for age, sex, education years, and marital status

P^*^ < 0.05

P^**^< 0.01

P^***^< 0.001

### Association of suicidal ideation, plan, and attempt with IA

The results of association of suicide ideation, plan, and attempt with IA are shown in Table 3, after dividing the subjects into 2 groups according to the presence of poor sleep quality. Participants with IA and sleep problems showed more frequent suicidal plan and suicidal attempt than participants with sleep problems but without IA. After adjusting for sex, age, education years, and marital status, suicide plan (AOR = 3.83, P = 0.005) and suicide attempt (AOR = 3.34, P = 0.007) showed a significant association with IA in the group with poor sleep quality. In participants free of sleep problems, IA showed a significant association with suicidal ideation (AOR = 2.17, P < 0.0001) and suicidal plan (AOR = 3.33, P = 0.013) after adjustment.

**Table 3:** Poor sleep quality and suicide ideation, plan, and attempt among adults with and without internet addiction (*n* = 3212).

Profiles	Internet addiction					
	Internet addiction (*n* = 204)		No internet addiction (*n* = 3008)			Statistics
	*N*	%	*N*	%	AOR	(95% CI)
With poor sleep quality						
1.Suicide ideation	18	23.4%	165	25.2%	1.02	(0.57–1.84)
2.Suicide plan	8	10.4%	38	5.8%	3.83	(1.52–9.68)^**^
3.Suicide attempt	9	11.8%	40	6.1%	3.34	(1.38–8.05)^**^
Without poor sleep quality						
1.Suicide ideation	27	21.8%	287	12.3%	2.17	(1.37–3.45)^***^
2.Suicide plan	6	4.8%	41	1.8%	3.33	(1.28–8.12)^*^
3.Suicide attempt	2	1.6%	42	1.8%	0.88	(0.20–3.80)

AOR, adjusted odds ratio; CI, confidence interval

Adjusted for age, sex, education years, and marital status

P* < 0.05

P**< 0.01

P***< 0.001

### Comorbidities of IA

The results of the logistic regression analysis of comorbidities in participants are shown in Table 4. After adjusting for age, sex, education years, and marital status, adults with both IA and sleep problems had significantly more frequent overall psychiatric disorders (AOR = 2.730, P = 0.001) and overall anxiety disorders (AOR = 10.088, P < 0.0001) than adults with IA but without sleep problems.

**Table 4:** Multiple logistic regression of comorbidities of Internet Addiction with poor sleep quality compared without poor sleep quality (n = 204).

DSM-IV disorders	Internet Addiction (*n* = 204)		Statistics		
	With Poor Sleep Quality (*n = 78*)	Without Poor Sleep Quality (*n* = 126)			
	N (%)	N (%)	AOR	(95% CI)	*P value*
MDD	8 (10.3)	12 (9.5)	0.804	(0.279–2.315)	0.686
Alcohol use disorder	32 (41.0)	30 (23.8)	2.384	(1.231–4.615)	0.010
Nicotine use disorder	15 (19.2)	14 (11.1)	2.063	(0.876–4.856)	0.097
Specific phobia	9 (11.5)	4 (3.2)	5.267	(1.340–20.704)	0.017
Any anxiety disorders	15 (19.2)	5 (4)	10.088	(2.842–35.811)	< .0001
Any DSM-IV disorders	50 (64.1)	51 (40.5)	2.730	(1.596–4.984)	0.001

AOR, adjusted odds ratio; CI, confidence interval

Adjusted for age, sex, education years, and marital status

## Discussion

Previous studies assessing IA usually evaluated its prevalence and phenomenology between adolescents and young adults [23, 40–43]. This is the first large cross-sectional study to investigate IA and sleep problems along with suicidal behavior in an adult population. Consequently, unique findings from this study showed specific demographic characteristics related to IA in the adult group such as younger age, more education, male gender, unmarried status and unemployment. Internet addiction was associated with poor sleep quality, which was shown as a problem in sleep initiation and maintenance, leading to non-restorative sleep, and daytime functional impairment. Participants with both IA and sleep problems showed increased risk of overall psychiatric disorder morbidity and suicide-related behavior.

Sleep disturbances related to IA have been reported in previous studies in the adolescent and young adult groups [41, 43], either as a consequence of IA [4, 43] related to anxiety or mood problems, or as a predictor of IA [41], depicted as disturbed circadian rhythm [41]. However, these studies assessing sleep disturbance related to IA demonstrated overall sleep quality severity including total sleep duration except for specific problems of poor sleep [41, 43, 44]. External factors such as exposure to the light source from the internet device, leading to suppression of melatonin secretion [45], and lack of physical activity that is important for restorative sleep [46] may help in explaining the results. Internal factors reported in other studies such as below-normal functioning of the dopaminergic system are the known risk factors for addiction [47, 48] and activation of sympathetic nervous tone in subjects with IA [49] may contribute to DIS, DMS and DFI. The association of several parasomnias with IA has been previously reported, but parasomnias, including sleep snoring, apnea, bruxism, and nightmares [10, 50] were not evaluated and they could be explainable factors for DMS. The specific result of this study such as ‘longer sleep time during the weekdays’ associated with IA could be an explanation for NRS and DFI in the IA group, and reverse results such as catch up on sleep on the weekends were associated with poor mental health [51]. Instead of simple application of IA diagnoses with corresponding duration of internet use, IAT including the related problematic behavior, craving, tolerance, withdrawal, and compulsive tendency, could have depicted its positive correlation with severe IA and severe sleep problems.

IA is a risk correlated with suicidal behavior [52–54], and even its limitation observed in the adolescent group was consistent with our findings, which can be explained by decreased cognitive control seen in neuroimaging studies in the IA group [42, 55]. On classifying suicide-related behavior into three different characteristics such as suicidal ideation, plan, and previous attempt, the current study showed that the IA with sleep problem group had more tendency for suicide plan and attempt, while the IA without sleep problem group had more tendency for suicide ideation and plan. Lack of the protective factor of storage sleep [27] in suicide, along with impulsivity seen in IA [25, 26] could have caused this difference.

The current study findings about comorbidities presented more frequent anxiety disorders and overall psychiatric comorbidities in subjects with IA accompanying sleep problems than in subjects with IA free from sleep problems. Considering high prevalence rate on anxiety disorder and IA from previous report [56], internet use not as complementary behavior to relieve depressed mood, but as compulsory behavior associated with anxiety could be suggested for this explanation. This also could be explained by the specific limitation of the IAT scale, which focuses on the severity with calculation of symptoms, and not on the specific purpose of internet use bringing lack of clarification about cause and relationship connected to comorbidities. The IAT scale has a blurred standard in internet devices, such as smartphone, tablet, and computer, and recently, a difference in the addictive characteristics was observed between the purpose and device [57].

## Methodological consideration

The first strong point of this study is that it is one of the few studies to examine and compare the correlation of sleep quality profiles and IA in the adult group. Second, it is the first study to examine the association between poor sleep quality and detailed suicidal behavior in subjects with IA. Third, this study applied the most popular IA scale, the IAT, and it showed a positive association between poor sleep quality and severity of IA.

However, some limitations of this study need to be brought forward. First, this is a cross-sectional study, using questionnaires and interview depending on subjective memory. Recall bias and limitations to objective measurement related to sleep may exist [58], and even a prior study, which reported that self-reported sleep patterns are similar to those obtained by wrist actigraphy [59], should be considered. Second, due to its cross-sectional research design, this study may not identify causal relationships between correlated characteristics and IA. Third, careful interpretation should be taken for Internet addiction as a diagnosis has not received widespread acceptance among the psychiatric community [60] and this study used the IAT to measure problematic internet use, omitting the specific content of internet use of the related device. These days, improvement in internet access has provided easy connection to internet, leading to emphasis on the purpose of use and characteristic differences in smartphone addiction and IA.

## Implication

The current study showed that a variety of poor sleep quality indexes were associated with IA and the association showed a positive correlation with IA severity. Considering the importance of adequate sleep in the protective effect on mental health and high risk of suicidal plan and attempt observed in the IA with sleep problem group, we suggest that health professionals should be aware of coexistence of sleep problems and IA, not only in the adolescent group but also in the adult group. Future studies should consider development of a scale containing information on the internet content and the device for access.

## References

[1] Wallace P. Internet addiction disorder and youth: There are growing concerns about compulsive online activity and that this could impede students' performance and social lives. EMBO reports. 2014;15(1):12–6. PubMed Central PMCID: PMC4303443. doi: 10.1002/embr.201338222 24398129 10.1002/embr.201338222 PMC4303443

[2] Shaw M, Black DW. Internet addiction: definition, assessment, epidemiology and clinical management. CNS drugs. 2008;22(5):353–65. 18399706 10.2165/00023210-200822050-00001

[3] Reed P, Vile R, Osborne LA, Romano M, Truzoli R. Problematic Internet Usage and Immune Function. PloS one. 2015;10(8):e0134538 PubMed Central PMCID: PMC4526519. doi: 10.1371/journal.pone.0134538 26244339 10.1371/journal.pone.0134538 PMC4526519

[4] Li W, O'Brien JE, Snyder SM, Howard MO. Characteristics of internet addiction/pathological internet use in U.S. university students: a qualitative-method investigation. PloS one. 2015;10(2):e0117372 PubMed Central PMCID: PMC4315426. doi: 10.1371/journal.pone.0117372 25647224 10.1371/journal.pone.0117372 PMC4315426

[5] Liu T, Potenza MN. Problematic Internet use: clinical implications. CNS spectrums. 2007;12(6):453–66. 17545956 10.1017/s1092852900015339

[6] Spada MM. An overview of problematic internet use. Addictive behaviors. 2014;39(1):3–6. doi: 10.1016/j.addbeh.2013.09.007 24126206 10.1016/j.addbeh.2013.09.007

[7] Dowling NA. Issues raised by the DSM-5 internet gaming disorder classification and proposed diagnostic criteria. Addiction. 2014;109(9):1408–9. doi: 10.1111/add.12554 25103097 10.1111/add.12554

[8] Bruni O, Sette S, Fontanesi L, Baiocco R, Laghi F, Baumgartner E. Technology Use and Sleep Quality in Preadolescence and Adolescence. Journal of clinical sleep medicine: JCSM: official publication of the American Academy of Sleep Medicine. 2015;11(12):1433–41. PubMed Central PMCID: PMC4661336. 26235161 10.5664/jcsm.5282 PMC4661336

[9] Cheung LM, Wong WS. The effects of insomnia and internet addiction on depression in Hong Kong Chinese adolescents: an exploratory cross-sectional analysis. Journal of sleep research. 2011;20(2):311–7. doi: 10.1111/j.1365-2869.2010.00883.x 20819144 10.1111/j.1365-2869.2010.00883.x

[10] Choi K, Son H, Park M, Han J, Kim K, Lee B, et al Internet overuse and excessive daytime sleepiness in adolescents. Psychiatry and clinical neurosciences. 2009;63(4):455–62. doi: 10.1111/j.1440-1819.2009.01925.x 19490510 10.1111/j.1440-1819.2009.01925.x

[11] Weinstein A, Lejoyeux M. New developments on the neurobiological and pharmaco-genetic mechanisms underlying internet and videogame addiction. The American journal on addictions / American Academy of Psychiatrists in Alcoholism and Addictions. 2015;24(2):117–25. 10.1111/ajad.12110 25864599

[12] Yau YH, Potenza MN. Gambling disorder and other behavioral addictions: recognition and treatment. Harvard review of psychiatry. 2015;23(2):134–46. PubMed Central PMCID: PMC4458066. doi: 10.1097/HRP.0000000000000051 25747926 10.1097/HRP.0000000000000051 PMC4458066

[13] Lai CM, Mak KK, Watanabe H, Jeong J, Kim D, Bahar N, et al The mediating role of Internet addiction in depression, social anxiety, and psychosocial well-being among adolescents in six Asian countries: a structural equation modelling approach. Public health. 2015;129(9):1224–36. doi: 10.1016/j.puhe.2015.07.031 26343546 10.1016/j.puhe.2015.07.031

[14] Kim NR, Hwang SS, Choi JS, Kim DJ, Demetrovics Z, Kiraly O, et al Characteristics and Psychiatric Symptoms of Internet Gaming Disorder among Adults Using Self-Reported DSM-5 Criteria. Psychiatry investigation. 2016;13(1):58–66. PubMed Central PMCID: PMC4701686. doi: 10.4306/pi.2016.13.1.58 26766947 10.4306/pi.2016.13.1.58 PMC4701686

[15] Weinstein A, Dorani D, Elhadif R, Bukovza Y, Yarmulnik A, Dannon P. Internet addiction is associated with social anxiety in young adults. Annals of clinical psychiatry: official journal of the American Academy of Clinical Psychiatrists. 2015;27(1):4–9. 25696775

[16] Yilmaz S, Herguner S, Bilgic A, Isik U. Internet addiction is related to attention deficit but not hyperactivity in a sample of high school students. International journal of psychiatry in clinical practice. 2015;19(1):18–23. doi: 10.3109/13651501.2014.979834 25356660 10.3109/13651501.2014.979834

[17] Perlis ML, Grandner MA, Chakravorty S, Bernert RA, Brown GK, Thase ME. Suicide and sleep: Is it a bad thing to be awake when reason sleeps? Sleep medicine reviews. 2015;29:101–7. doi: 10.1016/j.smrv.2015.10.003 26706755 10.1016/j.smrv.2015.10.003 PMC5070474

[18] Kay DB, Dombrovski AY, Buysse DJ, Reynolds CF, Begley A, Szanto K. Insomnia is associated with suicide attempt in middle-aged and older adults with depression. International psychogeriatrics / IPA. 2015:1–7. 10.1017/S104161021500174X PMC4808421 26552935

[19] Golding S, Nadorff MR, Winer ES, Ward KC. Unpacking Sleep and Suicide in Older Adults in a Combined Online Sample. Journal of clinical sleep medicine: JCSM: official publication of the American Academy of Sleep Medicine. 2015;11(12):1385–92. PubMed Central PMCID: PMC4661330. 26194726 10.5664/jcsm.5270 PMC4661330

[20] Woznica AA, Carney CE, Kuo JR, Moss TG. The insomnia and suicide link: toward an enhanced understanding of this relationship. Sleep medicine reviews. 2015;22:37–46. doi: 10.1016/j.smrv.2014.10.004 25454672 10.1016/j.smrv.2014.10.004

[21] Ko CH, Yen JY, Yen CF, Chen CS, Chen CC. The association between Internet addiction and psychiatric disorder: a review of the literature. European psychiatry: the journal of the Association of European Psychiatrists. 2012;27(1):1–8. 22153731 10.1016/j.eurpsy.2010.04.011

[22] Weinstein A, Lejoyeux M. Internet addiction or excessive internet use. The American journal of drug and alcohol abuse. 2010;36(5):277–83. doi: 10.3109/00952990.2010.491880 20545603 10.3109/00952990.2010.491880

[23] Lam LT. Internet gaming addiction, problematic use of the internet, and sleep problems: a systematic review. Current psychiatry reports. 2014;16(4):444 doi: 10.1007/s11920-014-0444-1 24619594 10.1007/s11920-014-0444-1

[24] Demirci K, Akgonul M, Akpinar A. Relationship of smartphone use severity with sleep quality, depression, and anxiety in university students. Journal of behavioral addictions. 2015;4(2):85–92. PubMed Central PMCID: PMC4500888. doi: 10.1556/2006.4.2015.010 26132913 10.1556/2006.4.2015.010 PMC4500888

[25] Yao YW, Wang LJ, Yip SW, Chen PR, Li S, Xu J, et al Impaired decision-making under risk is associated with gaming-specific inhibition deficits among college students with Internet gaming disorder. Psychiatry research. 2015;229(1–2):302–9. doi: 10.1016/j.psychres.2015.07.004 26168928 10.1016/j.psychres.2015.07.004

[26] Loh KK, Kanai R. How Has the Internet Reshaped Human Cognition? The Neuroscientist: a review journal bringing neurobiology, neurology and psychiatry. 2015. 10.1177/1073858415595005 26170005

[27] Germain A. Resilience and readiness through restorative sleep. Sleep. 2015;38(2):173–5. PubMed Central PMCID: PMC4288596. doi: 10.5665/sleep.4388 25581926 10.5665/sleep.4388 PMC4288596

[28] Cho MJ, Chang SM, Lee YM, Bae A, Ahn JH, Son J, et al Prevalence of DSM-IV major mental disorders among Korean adults: A 2006 National Epidemiologic Survey (KECA-R). Asian journal of psychiatry. 2010;3(1):26–30. Epub 2010/03/01. doi: 10.1016/j.ajp.2010.01.009 23051134 10.1016/j.ajp.2010.01.009

[29] World Health Organization. CIDI, Core Version 2.1 Trainer's Manual. Geneva: World Health Organization; 1997 1–244 p.

[30] World Health Organization. CIDI, Core Version 2.1 Interviewer's Manual. Geneva: World Health Organization; 1997 1–114 p.

[31] Cho MJ, Hahm BJ, Bae JN, Suh T, Lee DW, Cho SJ, et al Development of the Korean version of Composite International Diagnostic Interview. Annual Meeting of the Korean Neuropsychiatric Association. Seoul, Korea: Korean Neuropsychiatric Association; 1999.

[32] World Health Organization. Procedures for the Development of New Language Versions of the WHO Composite International Diagnostic Interview (WHO-CIDI). Geneva, Switzerland: World Health Organization; 1997.

[33] Cho MJ, Hahm BJ, Suh DW, Hong JP, Bae JN, Kim JK, et al Development of Korean Version of the Composite International Dianostic Interview(K-CIDI). J Korean Neuropsychiatr Assoc. 2002;41(1):123–37.

[34] Cho MJ, Kim JK, Jeon HJ, Suh T, Chung IW, Hong JP, et al Lifetime and 12-month prevalence of DSM-IV psychiatric disorders among Korean adults. The Journal of nervous and mental disease. 2007;195(3):203–10. Epub 2007/05/01. doi: 10.1097/01.nmd.0000243826.40732.45 17468679 10.1097/01.nmd.0000243826.40732.45

[35] Lee K, Lee HK, Gyeong H, Yu B, Song YM, Kim D. Reliability and validity of the Korean version of the internet addiction test among college students. Journal of Korean medical science. 2013;28(5):763–8. PubMed Central PMCID: PMC3653091. doi: 10.3346/jkms.2013.28.5.763 23678270 10.3346/jkms.2013.28.5.763 PMC3653091

[36] Young KS. Internet Addiction: The Emergence of an New Clinical Disorder CyberPsychology & Behavior. 1998;1(3):237–44.

[37] Jeon HJ, Roh MS, Kim KH, Lee JR, Lee D, Yoon SC, et al Early trauma and lifetime suicidal behavior in a nationwide sample of Korean medical students. Journal of affective disorders. 2009;119(1–3):210–4. Epub 2009/03/28. doi: 10.1016/j.jad.2009.03.002 19324420 10.1016/j.jad.2009.03.002

[38] Lee S, Fung SC, Tsang A, Liu ZR, Huang YQ, He YL, et al Lifetime prevalence of suicide ideation, plan, and attempt in metropolitan China. Acta Psychiatr Scand. 2007;116(6):429–37. Epub 2007/11/14. doi: 10.1111/j.1600-0447.2007.01064.x 17997722 10.1111/j.1600-0447.2007.01064.x

[39] Cho MJ, Hahm BJ, Suh DW, Hong JP, Bae JN, Cho SJ, et al A preliminary study for the 2006 National Survey of Psychiatric Illness. Seoul: Seoul National University,; 2005.

[40] Nie J, Zhang W, Chen J, Li W. Impaired inhibition and working memory in response to internet-related words among adolescents with internet addiction: A comparison with attention-deficit/hyperactivity disorder. Psychiatry research. 2016;236:28–34. doi: 10.1016/j.psychres.2016.01.004 26778632 10.1016/j.psychres.2016.01.004

[41] Chen YL, Gau SS. Sleep problems and internet addiction among children and adolescents: a longitudinal study. Journal of sleep research. 2016. 10.1111/jsr.12388 26854132

[42] Wen T, Hsieh S. Network-Based Analysis Reveals Functional Connectivity Related to Internet Addiction Tendency. Frontiers in human neuroscience. 2016;10:6 PubMed Central PMCID: PMC4740778. doi: 10.3389/fnhum.2016.00006 26869896 10.3389/fnhum.2016.00006 PMC4740778

[43] An J, Sun Y, Wan Y, Chen J, Wang X, Tao F. Associations between problematic internet use and adolescents' physical and psychological symptoms: possible role of sleep quality. Journal of addiction medicine. 2014;8(4):282–7. doi: 10.1097/ADM.0000000000000026 25026104 10.1097/ADM.0000000000000026

[44] Yen CF, Ko CH, Yen JY, Cheng CP. The multidimensional correlates associated with short nocturnal sleep duration and subjective insomnia among Taiwanese adolescents. Sleep. 2008;31(11):1515–25. PubMed Central PMCID: PMC2579980. 19014071 10.1093/sleep/31.11.1515 PMC2579980

[45] Higuchi S, Motohashi Y, Liu Y, Maeda A. Effects of playing a computer game using a bright display on presleep physiological variables, sleep latency, slow wave sleep and REM sleep. Journal of sleep research. 2005;14(3):267–73. doi: 10.1111/j.1365-2869.2005.00463.x 16120101 10.1111/j.1365-2869.2005.00463.x

[46] Bener A, Bhugra D. Lifestyle and depressive risk factors associated with problematic internet use in adolescents in an Arabian Gulf culture. Journal of addiction medicine. 2013;7(4):236–42. doi: 10.1097/ADM.0b013e3182926b1f 23666321 10.1097/ADM.0b013e3182926b1f

[47] Taber KH, Black DN, Porrino LJ, Hurley RA. Neuroanatomy of dopamine: reward and addiction. The Journal of neuropsychiatry and clinical neurosciences. 2012;24(1):1–4. doi: 10.1176/appi.neuropsych.24.1.1 22450608 10.1176/appi.neuropsych.24.1.1

[48] Kim SH, Baik SH, Park CS, Kim SJ, Choi SW, Kim SE. Reduced striatal dopamine D2 receptors in people with Internet addiction. Neuroreport. 2011;22(8):407–11. doi: 10.1097/WNR.0b013e328346e16e 21499141 10.1097/WNR.0b013e328346e16e

[49] Lu DW, Wang JW, Huang AC. Differentiation of Internet addiction risk level based on autonomic nervous responses: the Internet-addiction hypothesis of autonomic activity. Cyberpsychology, behavior and social networking. 2010;13(4):371–8. doi: 10.1089/cyber.2009.0254 20712495 10.1089/cyber.2009.0254

[50] Li S, Jin X, Wu S, Jiang F, Yan C, Shen X. The impact of media use on sleep patterns and sleep disorders among school-aged children in China. Sleep. 2007;30(3):361–7. 17425233 10.1093/sleep/30.3.361

[51] Kang SG, Lee YJ, Kim SJ, Lim W, Lee HJ, Park YM, et al Weekend catch-up sleep is independently associated with suicide attempts and self-injury in Korean adolescents. Comprehensive psychiatry. 2014;55(2):319–25. doi: 10.1016/j.comppsych.2013.08.023 24267542 10.1016/j.comppsych.2013.08.023

[52] Kaess M, Durkee T, Brunner R, Carli V, Parzer P, Wasserman C, et al Pathological Internet use among European adolescents: psychopathology and self-destructive behaviours. European child & adolescent psychiatry. 2014;23(11):1093–102. PubMed Central PMCID: PMC4229646. 24888750 10.1007/s00787-014-0562-7 PMC4229646

[53] Park S, Hong KE, Park EJ, Ha KS, Yoo HJ. The association between problematic internet use and depression, suicidal ideation and bipolar disorder symptoms in Korean adolescents. The Australian and New Zealand journal of psychiatry. 2013;47(2):153–9. doi: 10.1177/0004867412463613 23047959 10.1177/0004867412463613

[54] Husky MM, Michel G, Richard JB, Guignard R, Beck F. Gender differences in the associations of gambling activities and suicidal behaviors with problem gambling in a nationally representative French sample. Addictive behaviors. 2015;45:45–50. doi: 10.1016/j.addbeh.2015.01.011 25644586 10.1016/j.addbeh.2015.01.011

[55] Yuan K, Yu D, Cai C, Feng D, Li Y, Bi Y, et al Frontostriatal circuits, resting state functional connectivity and cognitive control in internet gaming disorder. Addiction biology. 2016. 10.1111/adb.12348 26769234

[56] Bernardi S, Pallanti S. Internet addiction: a descriptive clinical study focusing on comorbidities and dissociative symptoms. Comprehensive psychiatry. 2009;50(6):510–6. doi: 10.1016/j.comppsych.2008.11.011 19840588 10.1016/j.comppsych.2008.11.011

[57] Choi SW, Kim DJ, Choi JS, Ahn H, Choi EJ, Song WY, et al Comparison of risk and protective factors associated with smartphone addiction and Internet addiction. Journal of behavioral addictions. 2015;4(4):308–14. PubMed Central PMCID: PMC4712765. doi: 10.1556/2006.4.2015.043 26690626 10.1556/2006.4.2015.043 PMC4712765

[58] Knutson KL, Lauderdale DS. Sleep duration and overweight in adolescents: self-reported sleep hours versus time diaries. Pediatrics. 2007;119(5):e1056–62. doi: 10.1542/peds.2006-2597 17473079 10.1542/peds.2006-2597

[59] Wolfson AR, Carskadon MA, Acebo C, Seifer R, Fallone G, Labyak SE, et al Evidence for the validity of a sleep habits survey for adolescents. Sleep. 2003;26(2):213–6. 12683482 10.1093/sleep/26.2.213

[60] Byun S, Ruffini C, Mills JE, Douglas AC, Niang M, Stepchenkova S, et al Internet addiction: metasynthesis of 1996–2006 quantitative research. Cyberpsychology & behavior: the impact of the Internet, multimedia and virtual reality on behavior and society. 2009;12(2):203–7. 10.1089/cpb.2008.0102 19072075

